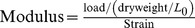# Polymorphisms in the Human Tropoelastin Gene Modify *In Vitro* Self-Assembly and Mechanical Properties of Elastin-Like Polypeptides

**DOI:** 10.1371/journal.pone.0046130

**Published:** 2012-09-25

**Authors:** David He, Ming Miao, Eva E. Sitarz, Lisa D. Muiznieks, Sean Reichheld, Richard J. Stahl, Fred W. Keeley, John Parkinson

**Affiliations:** 1 Program in Molecular Structure and Function, Hospital for Sick Children, Toronto, Ontario, Canada; 2 Department of Molecular Genetics, University of Toronto, Toronto, Ontario, Canada; 3 Department of Biochemistry, University of Toronto, Toronto, Ontario, Canada; Dalhousie University, Canada

## Abstract

Elastin is a major structural component of elastic fibres that provide properties of stretch and recoil to tissues such as arteries, lung and skin. Remarkably, after initial deposition of elastin there is normally no subsequent turnover of this protein over the course of a lifetime. Consequently, elastic fibres must be extremely durable, able to withstand, for example in the human thoracic aorta, billions of cycles of stretch and recoil without mechanical failure. Major defects in the elastin gene (*ELN*) are associated with a number of disorders including Supravalvular aortic stenosis (SVAS), Williams-Beuren syndrome (WBS) and autosomal dominant cutis laxa (ADCL). Given the low turnover of elastin and the requirement for the long term durability of elastic fibres, we examined the possibility for more subtle polymorphisms in the human elastin gene to impact the assembly and long-term durability of the elastic matrix. Surveys of genetic variation resources identified 118 mutations in human *ELN*, 17 being non-synonymous. Introduction of two of these variants, G422S and K463R, in elastin-like polypeptides as well as full-length tropoelastin, resulted in changes in both their assembly and mechanical properties. Most notably G422S, which occurs in up to 40% of European populations, was found to enhance some elastomeric properties. These studies reveal that even apparently minor polymorphisms in human *ELN* can impact the assembly and mechanical properties of the elastic matrix, effects that over the course of a lifetime could result in altered susceptibility to cardiovascular disease.

## Introduction

Elastin is a polymeric extracellular protein responsible for imparting properties of extensibility and elastic recoil to various tissues found in vertebrate organisms. In humans, elastin is a key component of arterial tissues with, for example, the thoracic aorta being composed of up to 50% elastin by dry weight [1]. Elastin is produced as a soluble monomer called tropoelastin. Once secreted, it polymerizes, along with other protein components, into elastic fibres that together form an extracellular elastic matrix whose structural integrity is crucial for the mechanical stability and physical properties of the tissue [2].

A critical step in polymer formation is the self-assembly of tropoelastin into a matrix through a process of temperature-induced phase separation known as coacervation [3], [4]. The ability of elastin to assemble into a polymer and the functional properties of the elastic matrix are dependent on its protein sequence. The human tropoelastin gene is composed of 34 exons, resulting in a protein with an alternating arrangement of hydrophobic and cross-linking domains. The former are rich in glycine, proline, alanine, leucine and valine, often organized in short (3–9 amino acids) tandemly repeated sequences forming flexible and highly dynamic structures, including short beta-sheet/beta-turn conformers. These regions are believed to impart elastomeric properties to elastin by a primarily entropic mechanism. The cross-linking domains in human elastin are shorter, and generally rich in alanines interspersed with the lysine residues that are used to form covalent crosslinks to stabilize the elastin polymer. These alanine-rich crosslinking domains are predicted to be at least partially alpha-helical in nature [2], [5].

Given the importance of elastin in the aorta and other elastic arteries, it is not surprising that disorders in elastin production and assembly can result in cardiovascular disease. Known genetic diseases associated with elastin include the haploinsufficiency disorders, supravalvular aortic stenosis (SVAS) and Williams-Beuren syndrome (WBS), with cardiovascular consequences [5]–[7]. The autosomal dominant variant of cutis laxa (ADCL), with a phenotype mainly involving skin and lung tissues, arises from missense mutations altering the protein sequence of the final few domains of human tropoelastin, including the C-terminal exon thought to be important for elastic fibre assembly [5], [6], [8]. Aortic elastin is unusual in that, once laid down during neonatal development, the protein remains with little or no subsequent turnover [9], [10]. Given that the human aorta must undergo billions of cycles of stretch and recoil over a normal lifetime, the elastic matrix must be remarkably durable. As a consequence it has been speculated that polymorphisms in the elastin gene resulting in alterations in the amino acid sequence of elastin could lead to subtle changes in the architecture of the elastic fibre, in the long-term rendering these elastic fibres more prone to failure.

To explore this hypothesis, and to examine the potential impact of sequence mutations on the functional and mechanical properties of elastin, we have undertaken a survey of publicly available human single nucleotide polymorphisms (SNPs) to identify non-synonymous mutations in regions of the elastin gene thought to play critical roles in determining the properties of elastin [11], [12]. Using polypeptides previously shown to model elastin assembly and mechanics [13]–[18] as well as variants of full-length tropoelastin, we demonstrate that these polymorphisms can significantly affect normal *in vitro* self-assembly, and modify the mechanical properties of an *in vitro* crosslinked polymeric elastin matrix. These studies represent a novel approach to understanding sequence-function-phenotype relationships for important cardiovascular proteins such as elastin.

## Results

### Identification of non-synonymous polymorphisms in human tropoelastin

**Figure 1:**
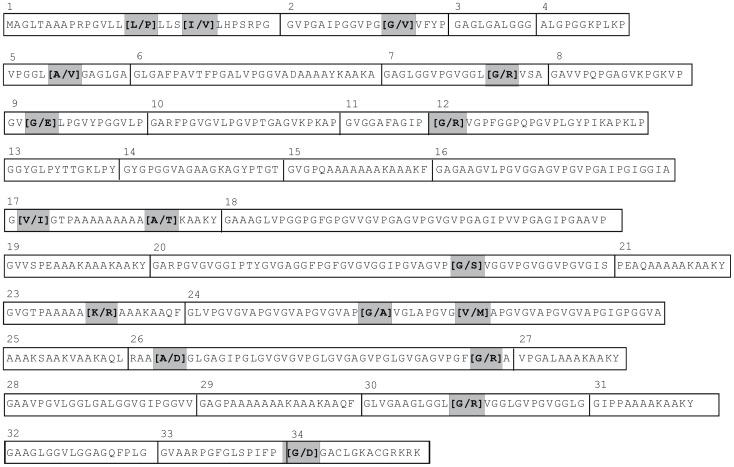
Single nucleotide polymorphisms causing non-synonymous changes in tropoelastin. Tropoelastin protein sequence corresponds to RefSeq, variant 1 (NM_000501). This variant does not include exons 22 or 26a (an extension of exon 26). Exons are boxed, with exon numbers above. The positions of mutations and substituted amino acids are indicated within the shaded boxes, minor allele is indicated second.

From the dbSNP database (http://www.ncbi.nlm.nih.gov/SNP), 110 single nucleotide polymorphisms (SNPs) were identified to be associated with the tropoelastin gene, of which 16 were located in exons. Twelve of these were non-synonymous, resulting in changes in amino acid sequence. A further eight potential exonic SNPs were identified from analysis of expressed sequence tags, five of which resulted in non-synonymous polymorphisms. Of this total of 17 SNPs (Figure 1 and Table 1), over half (9/17) resulted in a glycine substitution. This was not surprising given the abundance of glycine (30%) in the tropoelastin protein. However, no SNPs resulting in proline mutations were apparent, despite the substantial proportion of proline (12%) in tropoelastin. This observation may reflect the important role of prolines in initiating β-turns, a structural feature thought to be crucial for assembly and elastomeric properties of the elastic matrix [12], [19]–[21]. Indeed, previous studies replacing prolines with glycines in hydrophobic domains of elastin-like polypeptides abolished their ability to coacervate, and promoted formation of amyloid-like fibrils [16].

**Table 1:** Single nucleotide polymorphisms identified in human tropelastin.

SNP #	dbSNP reference^1^	Exon #	mRNA Position^2^	NucleotideSubstitution^3^	Amino AcidPosition^2^	AminoAcid Substitution^3^	SequenceContext^4^	Minor allele frequency(%)^5^
1	EST	1	47	T/C	16	Leu/Pro	VLL[P]LLS	0.5
2	EST	1	58	A/G	20	Ile/Val	LLS[V]LHP	0.5
3	rs55951999	2	119	G/T	40	Gly/Val	VPG[V]VFY	0–2.2
4	rs41350445	5	212	C/T	71	Ala/Val	GGL[V]GAG	2.0
5	EST	7	364	G/A	122	Gly/Arg	GGL[R]VSA	1.4
6	EST	9	434	G/A	145	Gly/Glu	PGV[E]LPG	0.5
7	EST	11^6^	571	G/A	191	Gly/Arg	GIP[R]VGP	4.0
8	rs41526244	17	892	G/A	298	Val/Ile	IAG[I]GTP	0.5–2.6
9	rs41376344	17	931	G/A	311	Ala/Thr	AAA[T]KAA	2.0
10	rs2071307	20	1264	G/A	422	Gly/Ser	GVP[S]VGG	9.0–43
11	rs34945509	23	1388	A/G	463	Lys/Arg	AAA[R]AAA	0.6–1.8
12	rs56307747	24	1481	G/C	494	Gly/Ala	VAP[A]VGL	0–2.2
13	rs41523046	24	1507	G/A	503	Val/Met	GVG[M]APG	4.0
14	rs34018370	26	1631	C/A	544	Ala/Asp	RAA[D]GLG	N/A
15	rs17855988	26	1741	G/A	581	Gly/Arg	PGF[R]AVP	2.1–8.7
16	rs34852121	30	1951	G/A	651	Gly/Arg	GGL[R]VGG	1.3
17	rs41511151	34	2131	G/A	711	Gly/Asp	IFP[D]GAC	0.3–2.0

1. SNPs identified through dbSNP are indicated with an appropriate SNP reference. EST indicates that the polymorphism was identified through EST libraries.

2. Tropoelastin RefSeq variant 1 (NM_000501) was used for numbering mRNA and amino acid positions, counting from the initiator methionine. Exons 22 and 26a (an extension of exon 26) are not present in this variant and are not included in the position count.

3. T/C and Leu/Pro designate Major/Minor allele respectively e.g. from T to C or Leu to Pro, etc.

4. Minor allele amino acid is indicated in square brackets

5. For SNPs detected through ESTs, minor allele frequency (MAF) indicates the proportion of ESTs sequences coding the minor allele. For SNPs obtained from dbSNP, MAF indicates the range across populations provided through dbSNP.

6. Because of phase 1 intron/exon borders, although the mutation site is in the last base of exon 11 the mutated amino acid is the first amino acid coded by exon 12.

Of these 17 SNPs, four (SNPs # 2, 4, 8 and 12) could be considered to result in conservative substitutions, less likely to affect the overall properties of the protein. However, other SNPs appeared to have the potential to affect protein production, structure or properties. For example, SNP #1 introduces a proline residue into the polyleucine motif of the signal peptide, possibly affecting signal processing and secretion of the protein. Furthermore, two of these SNPs (#s 7 and 10) modify -V(I)PG- motifs, which have been identified as common and abundant in tropoelastins over a broad phylogenetic range, and are likely involved in the initiation of the important β-turn structures in the protein [12]. In addition, several SNPs (#s 5, 6, 14, 15, and 16) introduce charged residues into characteristically non-polar domains. Particularly notable is SNP # 6 in which glutamic acid is substituted for a glycine in a hydrophobic domain, the sequence of which is strongly conserved across all species [11]. Similarly, SNP # 13 substitutes a methionine for a valine residue, notable because methionine residues, other than the initiator methionine at the N-terminal of the signal peptide, are characteristically absent from all mammalian and reptilian elastins, and extremely rare in elastins of other species [11]. Two SNPs (#s 9 and 11) affect the sequence of crosslinking domains, in one case (SNP #11) removing a crosslinking lysine residue. Finally, SNP #17, introduces a charged residue into a C-terminal exon sequence otherwise highly conserved over a broad phylogenetic range, and clearly associated with *in vivo* assembly of elastic fibres [5], [11], [22].

### Generation of recombinant elastin-like polypeptides (ELPs) incorporating identified SNPs

Although, as indicated above, several of these polymorphisms had the potential to significantly affect functional properties of human tropoelastin, SNPs 10 and 11 were chosen for modeling in recombinant elastin-like polypeptides because both introduced mutations to otherwise well-conserved sequence motifs. Furthermore, both the self-assembly behaviour of the monomer and the physical properties of the crosslinked polymer form of ELPs containing the domains involving these polymorphisms (domains 20 and 23) had already been well-characterized in our laboratory [13]–[17]. The sequences and domain arrangements of the ELPs used in this study are shown in Tables 2 and 3. Our original expectation was that since these effects appear in the human population, their impact on function may be relatively subtle. We therefore decided to attempt to accentuate the effect (if any) of the selected mutations by introducing multiple copies in the ELPs. As indicated, two ELPs mimicking the K to R substitution in domain 23 (SNP #11) were produced, containing this mutation in either one or both of the copies of domain 23 in this polypeptide. Similarly, two ELPs introducing the G to S substitution in domain 20 (SNP #10) were produced. One of these mimicked the single site of mutation present in the SNP, while the second introduced two additional instances of G to S substitutions in -VPG- motifs in domain 20.

**Table 2:** Domain Sequences Represented in Elastin-Like Polypeptides.

Domain Name	Type	Sequence
20	hydrophobic	FPGFGVGVGGIPGVAGVPGVGGVPGVGGVPGVGIS
20[1G/1S](SNP#10)	mutated hydrophobic	FPGFGVGVGGIPGVAGVP**S**VGGVPGVGGVPGVGIS
20[3G/3S]	mutated hydrophobic	FPGFGVGVGGIPGVAGVP**S**VGGVP**S**VGGVP**S**VGIS
24	hydrophobic	GLVPGVGVAPGVGVAPGVGVAPGVGLAPGVGVAPGVGVAPGVGVAPAIGP
21	crosslinking	PEAQAAAAAKAAKY
23	crosslinking	GVGTPAAAAAKAAAKAAQF
23[K/R](SNP#11)	mutated crosslinking	AAAAAAAAAAKAAA**R**AAQF

Mutated amino acids are indicated in bold

**Figure 2:**
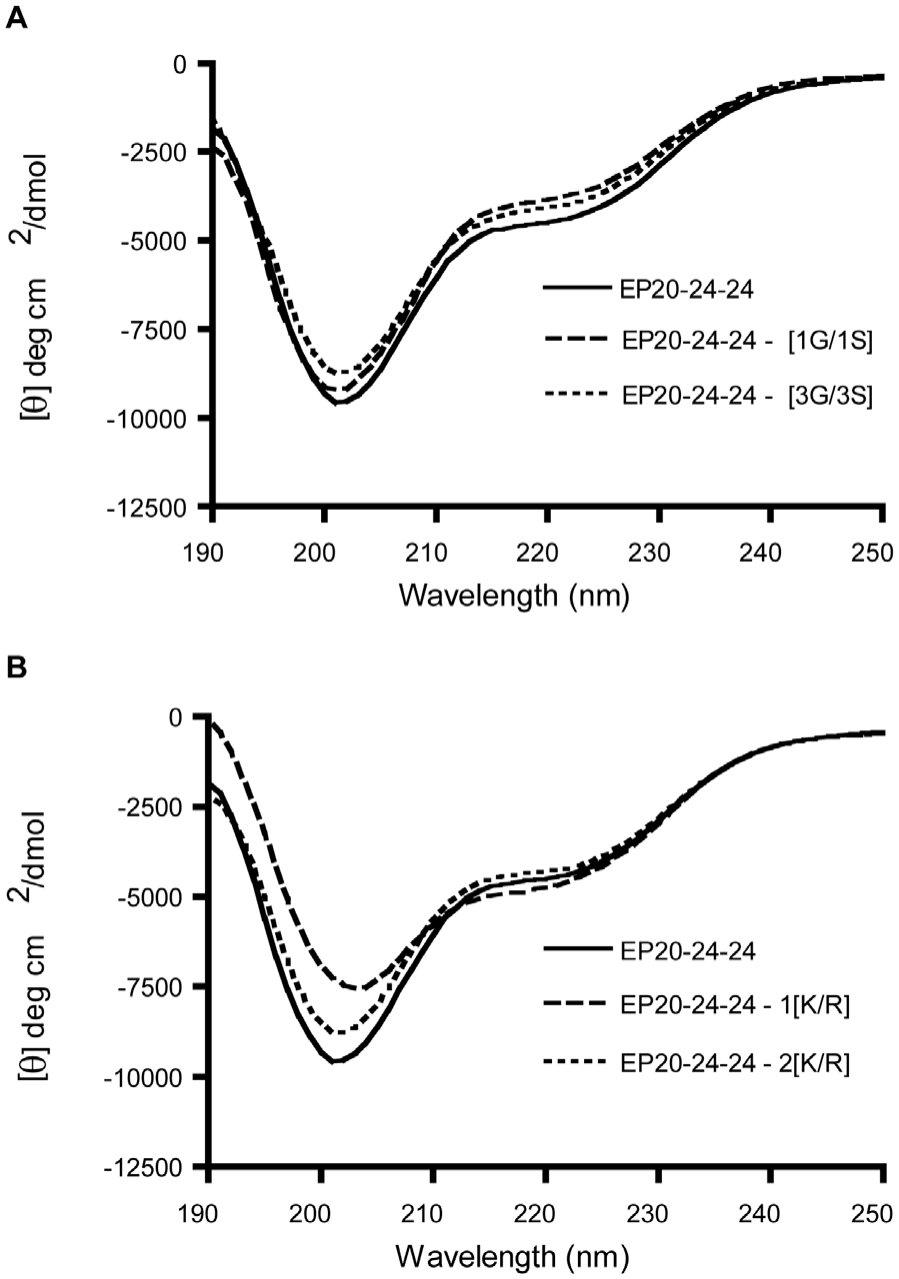
Effect of introducing select amino acid substitutions on the secondary structure of elastin-like peptides (ELPs). (A) CD spectra comparing reference ELP, EP20–24–24, with ELPs containing single and triple G to S substitutions. (B) CD spectra comparing reference ELP, EP20–24–24, with ELPs containing single and double K to R substitutions. Compared to the reference polypeptide, the introduction of the substitutions does not appear to result in any major changes in conformation of these ELPs.

Introduction of these mutations into the reference ELP (EP20–24–24) did not appear to result in any major changes in conformation of these ELPs, as determined by circular dichroism (CD) spectroscopy (Figure 2). Although some small spectral differences were apparent, in all cases these ELPs showed a propensity for disordered structure (large negative peak at about 200 nm) usually associated with the hydrophobic domains together with the limited α-helical content (minor negative peak at 222 nm) associated with crosslinking domains, characteristic of these ELPs as well as full-length tropoelastin [3]. These results suggested that the general secondary structural properties of these ELPs were not altered significantly by any of the mutations.

### Effect of mutations on self-assembly properties of ELPs

Coacervation is considered to be an important first step in the assembly of the elastic fibre *in vivo* [23], [24]. Studies focused on ELPs as a model for coacervation, indicate a two-step process, beginning with a temperature-induced phase separation resulting in the formation of a colloidal suspension of small, protein-rich droplets known as a coacervate. The temperature at which this transition takes place is dependent both on solution conditions, including salt and polypeptide concentrations, as well as the nature of the ELP, including molecular weight and amino acid sequence [14], [16], [17]. This coacervation step is reversible; if the suspension is cooled immediately the droplets will dissociate back into solution [25]. However, if the turbid suspension is held above the transition temperature an irreversible maturation process begins, corresponding to a decrease in turbidity [15], [26]. The progress and rate of the coacervation and maturation steps can be followed by monitoring absorbance at 440 nm [15], [26].

**Figure 3:**
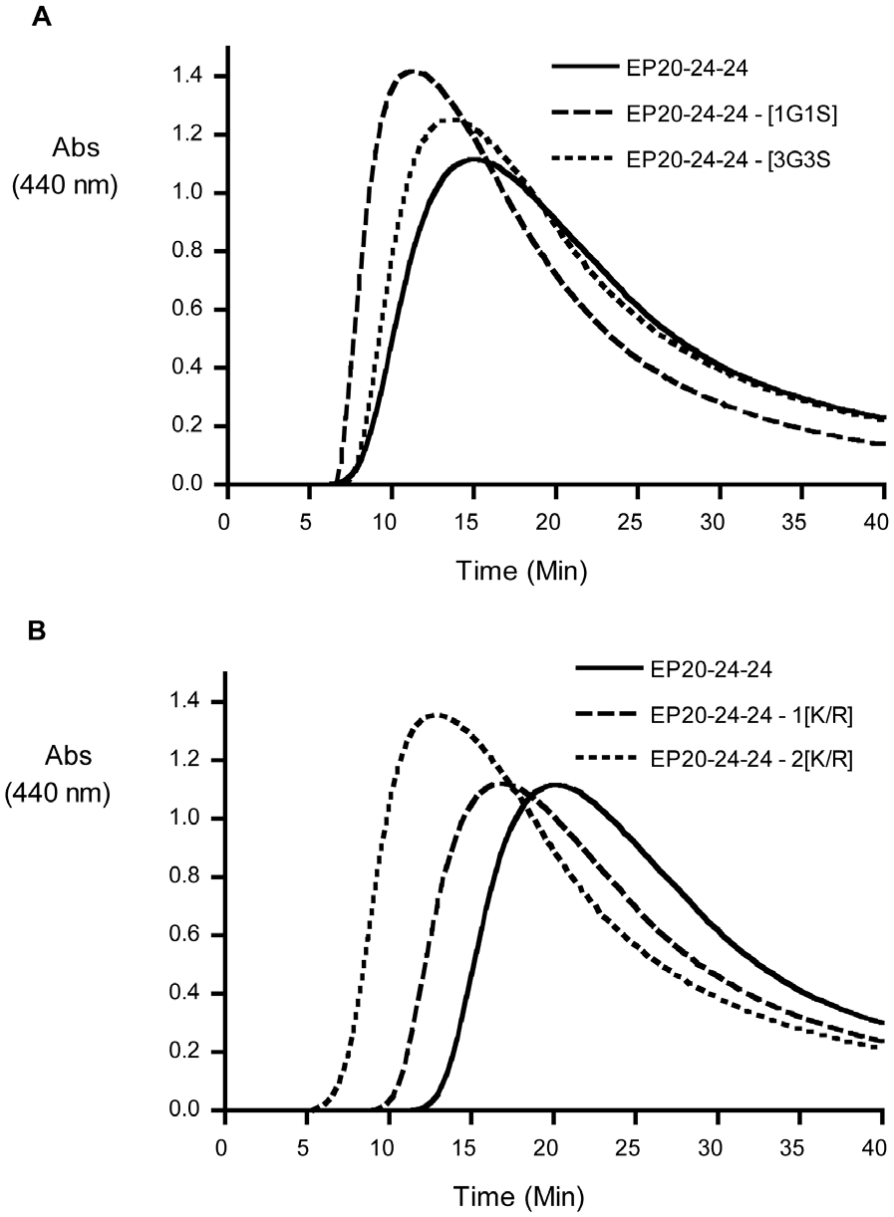
Coacervation characteristics of select elastin-like peptides (ELPs). (A) Coacervation (temperature-induce phase separation) of reference ELP, EP20–24–24, and ELPs containing single and triple G to S substitutions. Time 0 corresponds to 20°C, and temperature was raised at a rate of 1°C/min. (B) Coacervation of reference ELP, EP20–24–24, and ELPs containing single and double K to R substitutions. Time 0 corresponds to 15°C, and temperature was raised at a rate of 1°C/min. Coacervation was followed by turbidity as measured by absorbance at 440 nm. Curves represent means for three replicate experiments. Note the curves for the K to R substitutions are shifted to the left indicating that coacervation is initiated at a lower temperature.

Compared to the reference ELP (EP20–24–24), neither single nor multiple glycine to serine mutations showed any significant effect on the temperature at which coacervation was initiated (Tc) (Table 3). Similarly, these substitutions had no effect on the general shape of the coacervation curve (Figure 3A). Furthermore, neither single nor mutiple G to S mutations introduced into domain 20 had any effect on coacervation temperatures or the shape of the overall coacervation curves of full-length tropoelastin variants (data not shown).

**Table 3:** Characteristics of Elastin-Like Polypeptide

Designation	Domain Arrangement	Mol Wt(Da)	K-D Hydrop	Tc (mean±SD)(°C)
EP20–24–24	20–21–23–24–21–23–24	16,999	0.92	27.6±0.56 ^3^
EP20–24–24 – 1[K/R]	20–21–23[K/R]–24–21–23–24	17,020	0.92	25.4±0.45 ^3,4^
EP20–24–24 – 2[K/R]	20–21–23[K/R]–24–21–23[K/R]–24	17,048	0.92	22.2±0.15 ^3,4^
EP20–24–24 – [1G/1S]	20[1G/1S]–21–23–24–21–23–24	17,022	0.92	26.6±0.21 ^3^
EP20–24–24 – [3G/3S]	20[3G/3S]–21–23–24–21–23–24	17,082	0.92	27.8±0.31 ^3^
hTE - [20:G]	Full-length human tropoelastin (NM_000501) with G in amino acid position 422^1^	59,930	0.70	n/a
hTE - [20:S]	Full-length human tropoelastin (NM_000501) with S in amino acid position 422^1^	59,960	0.70	n/a
hTE - [20:3S]	Full-length human tropoelastin (NM_000501) with S in amino acid positions 422, 428 and 434^2^	60,020	0.70	n/a

1. See Figure 1

2. See Table 2

3. Coacervation conditions: 25 µM polypeptide, 1.5 M NaCl

4. p<0.01 vs. EP20–24–24 (n = 3)

In contrast, ELPs containing lysine to arginine mutations in one or both copies of crosslinking domain 23 showed a small but significant decrease in coacervation temperature (Table 3), again with little effect on the general shape of the coacervation curve (Figure 3B). This change in coacervation temperature was seen in spite of little or no change in either the molecular weight or the average hydropathy of the ELP (Tab1e 3), both of which factors have been shown to affect coacervation temperature [14], [16], [17].

### Effect of mutations on elastomeric properties of materials produced from ELPs

We have previously shown that coacervation of ELPs of the types used in this study results in an alignment of lysine residues in the crosslinking domains, allowing lysine-based crosslinking and fabrication of materials whose mechanical properties can be measured [13], [18]. In this case genipin, a short-arm glycation crosslinker was used to bridge between juxtaposed lysine residues [18], [27]–[29]. Elastomeric properties, including modulus, strain-to-break, energy loss per cycle and stress relaxation were measured for materials made from several of these ELPs, as well as the variants of full-length human tropoelastin containing single or multiple G to S substitutions.

**Figure 4:**
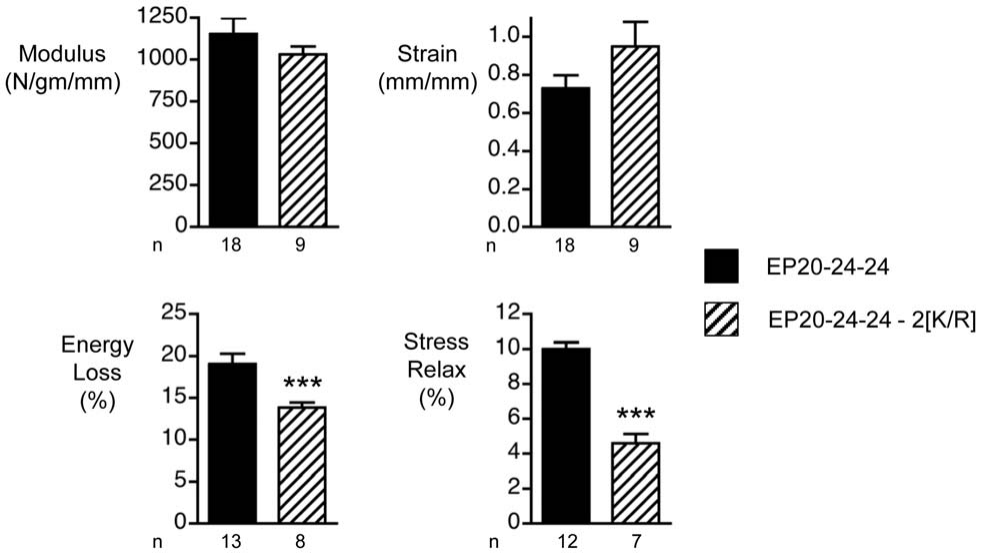
Mechanical properties of elastin-like peptides (ELPs) containing K to R substitutions. The four bar graphs indicate the means and standard errors for tensile mechanical properties of sheets of materials constructed with reference ELP, EP20–24–24, and ELPs containing the double K to R substitution. The number of replicates for each experiment (n) is indicated. *** indicates a significant difference between the two materials (ANOVA with Bonferoni correction, p<0.005).

In the case of the K to R substitution, only materials made from the ELP incorporating the mutation in both copies of domain 23 was tested for mechanical properties (Figure 4). Compared to materials made from the reference polypeptide (EP20–24–24), no significant differences in modulus or strain-to-break were evident. However, both % energy loss and % stress relaxation parameters were significantly decreased as a result of the K to R substitution in both copies of domain 23. This is an unexpected result, since removal of potential crosslinking sites would not be anticipated to result in such changes which might be regarded as an improvement in elastomeric properties.

**Figure 5:**
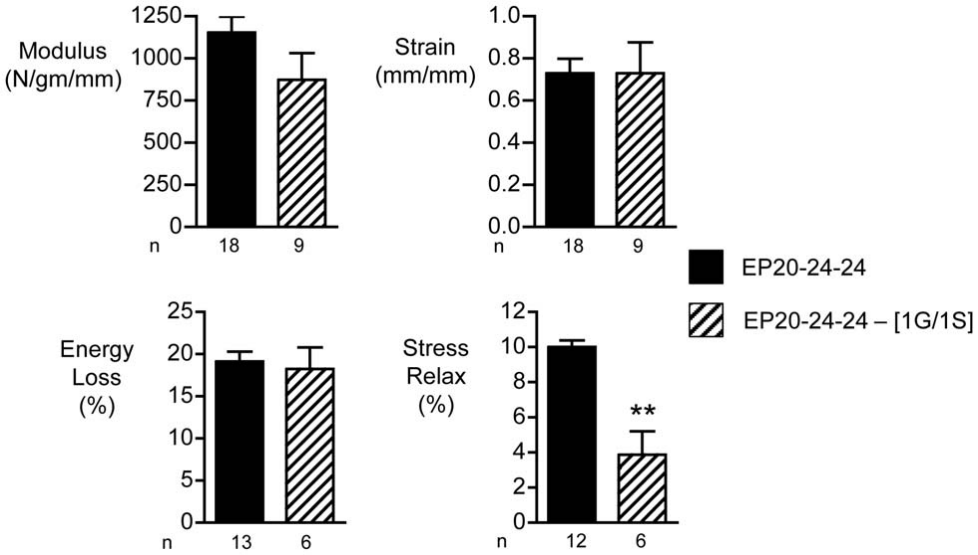
Mechanical properties of elastin-like peptides (ELPs) containing G to S substitutions. The four bar graphs indicate the means and standard errors for tensile mechanical properties of sheets of materials constructed with reference ELP, EP20–24–24, and ELPs containing the single G to S substitution. Materials constructed from ELPs containing the triple G to S substitution were too fragile to generate meaningful values. The number of replicates for each experiment (n) is indicated. ** indicates a significant difference between the two materials (ANOVA with Bonferoni correction, p<0.01).

Similarly, a single G to S substitution in domain 20 of the ELP produced no detectable change in modulus or strain-to-break properties (Figure 5). In addition, % energy loss was also unchanged, although there was a significant decrease in % stress relaxation as a result of this substitution. In contrast, when three G to S substitutions were introduced into the ELP, the materials formed had no structural integrity, and either could not be mounted for testing or immediately broke on initial extension, suggesting an interference with the crosslinking process.

**Figure 6:**
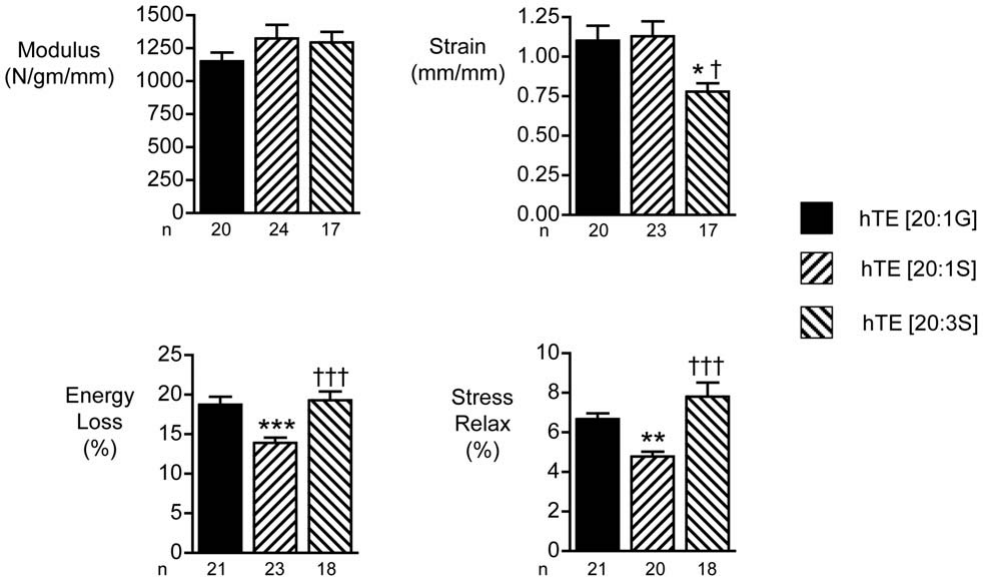
Mechanical properties of full-length human tropoelastin containing G to S substitutions. The four bar graphs indicate the means and standard errors for tensile mechanical properties of sheets of materials constructed with full-length human tropoelastin (hTE) and hTE variants containing either a single or triple G to S substitution in domain 20. The number of replicates for each experiment (n) is indicated. *, ** and *** indicate a significant difference between hTE and hTE with the single G to S substitution (ANOVA with Bonferoni correction, p<0.05, p<0.01 and p<0.001 respectively). † and ††† indicate a significant difference between hTE with the single G to S substitution and hTE with the triple G to S substitution (ANOVA with Bonferoni correction, p<0.05, p<0.001 respectively).

Because of the significant effects of the G to S substitutions on the material properties of ELPs and the relatively high incidence of this mutation in the human population, we investigated the effect on elastomeric properties of the single and triple G to S substitutions in domain 20 of full-length tropoelastin (Figure 6). In this case, human tropoelastin containing G at position 422 in the protein sequence was used as the reference material. A single G to S substitution at position 422 resulted in no detectable change in modulus or strain-to break properties. However, this mutation resulted in a significant reduction in both % energy loss and% stress relaxation in the material. Although the introduction of three G to S substitutions in domain 20 of full-length human tropoelastin did not result in the dramatic loss of structural integrity seen in ELPs, nevertheless this triple mutation both significantly reduced the strain-to-break parameter, and resulted in reversal of the improvements in % energy loss and % stress relaxation parameters that were seen for the single mutation.

## Discussion

Previously identified heritable disorders of elastin result from significant mutations in the form of either gene deletions, resulting in haploinsufficiency (SVAS and WBS) [5]–[7] or loss of a critical C-terminal exon required for integration into the extracellular matrix (ADCL) [5], [6], [8]. Given the longevity of elastin in the human aorta, we were interested in exploring the hypothesis that more subtle mutations in human *ELN* result in minor changes in assembly and/or mechanical properties. While these changes would not be expected to have an observable physiological consequence in the short term, due to the low turnover of the elastic matrix, they could, over the course of an individual's lifetime, impact susceptibility to degradative influences and thus result in an increased risk to later-onset cardiovascular disease. This hypothesis is supported by studies linking mutations in elastin with more complex multifactorial manifestations of cardiovascular disease [30], [31]. For example, several genetic association studies have linked elastin with the development of intracranial aneurysms (IA) [30], [32], [33] as well as increased risk of isolated systolic hypertension [34] and age-related alterations in carotid artery distensibility [35].

From population genetic datasets we identified 118 SNPs, of which 17 represent exonic non-synonymous mutations. We selected two of these for functional characterization on the basis of their occurrence within otherwise well-conserved sequence motifs that had already been modeled in recombinant ELPs [13]–[18]. G422S occurs in a repetitive VPG sequence in domain 20, an example of a conserved motif that is thought to initiate the formation of β-turn structures in the hydrophobic domains of the protein [12]. K463R is a residue involved in forming the cross-links that stabilize the polymeric matrix. To exaggerate the effects of these relatively conservative substitutions, both single and multiple copies of these substitutions were introduced into ELPs. None of these substitutions appeared to impact overall secondary structural properties. This is consistent with previous studies which demonstrated significant changes in CD spectra required more drastic substitutions such as the replacement of all prolines with glycines in domain 24 [16]. However, it is important to note that analyses of CD spectra provide only limited resolution, describing secondary structure only in a global and qualitative manner.

Coacervation studies found that while G to S substitutions did not affect assembly properties, K to R substitutions reduced the temperature of coacervation. Previous work has shown the propensity to coacervate is related, at least in part, to overall hydrophobicity and molecular weight [16], both of which were unaffected by these substitutions. On the other hand, in the case of the K to R substitution, local changes in hydrophobicity caused by the replacement of the lysine residue with a more hydrophobic arginine residue appeared to be sufficient to result in a small but measurable decrease in coacervation temeperature for the mutated ELP. Although decreased coacervation temperature of tropoelastin and ELPs has been associated with increased α-helical content, this is an unlikely explanation since a decrease in coacervation temperature of 5^°^C in Tc would require a doubling of α-helical content [36], an increase not supported by our CD data for these ELPs.

Mechanical studies revealed that the introduction of G to S and K to R substitutions in ELPs and G to S substitutions in full-length tropoelastin, resulted in materials with altered mechanical properties. While a single G to S substitution at position 422 significantly reduced both % energy loss and % stress relaxation, the triple G to S substitution resulted in an increase in these properties in full-length tropoelastin and loss of structural integrity in the ELP. Current models propose that as elastin is stretched, exposure of hydrophobic amino acids to the surrounding solvent results in decreased entropy, providing a driving force for the return to the relaxed state [37]–[39]. For the single G to S substitution, the observed beneficial effects might therefore arise from local structural changes in the domain that contribute to a larger decrease in entropy upon extension. Interestingly, in certain human populations (e.g. those of European ancestry), the allele responsible for the G to S substitution (dbSNP reference: rs2071307) occurs with a frequency as high as 40%. Our findings suggest that carriers of this allele might therefore expect improved performance from their elastin bearing tissues, offering an explanation for the high frequency associated with this allele. On the other hand, the catastrophic loss in structural integrity associated with the triple substitution in the ELP suggests a dramatic change in local structure, possibly through significant realignment of neighbouring cross-linking domains [40]. Introduction of the triple substitution in the full length protein would also be expected to alter local structure, however the dramatic effect observed in the ELP would be moderated by the presence of many additional domains that partially rescue the correct assembly architecture and crosslink formation.

For the K to R double substitutions introduced into the ELP, we again noted a significant decrease in both % energy loss and % stress relaxation parameters. This somewhat surprising result may be a consequence of using genipin, as opposed to the *in vivo* cross-linker lysyl oxidase (LOX) for which *in vitro* use is problematic [41]. Amino acid analysis of the cross-linked material confirmed that the arginine residues are not involved in cross-links formed by genipin (data not shown). However, formation of the principal crosslinks in elastin, desmosine and isodesmosine, by LOX is a complex process ultimately requiring alignment of four lysine residues. In contrast, genipin crosslinking requires proximity of only two lysine residues and may be less likely to be affected by mutation of a single lysine. Thus, *in vivo* crosslinking of elastin by LOX and the consequent material properties of the polymer might be expected to be more sensitive to this K to R substitution.

Together, these studies highlight the interdependence of hydrophobic and crosslinking sequences during assembly and for imparting mechanical properties. While crosslinking arises from lysine residues and hydrophobic sequences provide the predominant structural disorder that drives entropic elastic recoil [21], [25], [42], [43], a single amino acid substitution in one type of domain clearly has the potential to influence the assembly architecture and/or mechanical function of the resulting mature material. Our study highlights two key mutations in elastin worthy of further investigation. Of special interest is the proposed impact of the G to S substitution on entropic changes during extension of the protein, a hypothesis that is particularly amenable to study through established molecular dynamic simulations [21], [38].

These results suggest that even relatively subtle mutations may significantly impact the assembly and mechanical properties of human elastin. Given the longevity of the protein, such defects have the potential, over the course of a lifetime, to alter susceptibility to cardiovascular disease. Due to their manifestation only later in life, evolutionary pressure against such defects would be minimal. With population studies focused on specific cohorts of individuals diagnosed with cardiovascular disease [44], [45], opportunities now exist to identify and characterize additional rare genetic variants associated with the elastin gene.

## Materials and Methods

### Identification of Elastin Polymorphisms

Publicly available SNPs were acquired from two main sources: dbSNP (http://www.ncbi.nlm.nih.gov/projects/SNP/) [46], and publicly available expressed sequence tags (ESTs) from dbEST (http://www.ncbi.nlm.nih.gov/dbEST/) [47]. SNPs were retrieved from dbSNP by performing a search for the human elastin gene (ELN).

The identification of SNPs using existing EST datasets began with the collection of elastin ESTs using a BLAST search of the coding region of the human elastin gene (gi|152112965) against all publicly available human ESTs. In all, 500 ESTs were obtained, most of which were associated with the 5′ end of the coding region covering exons 1 to 15. ESTs were assembled into a consensus sequence using the software package phrap (http://www.phrap.com/). The resulting consensus sequence was 2041bp long. The absence of an abundant collection of ESTs outside the 5′ end of the coding region of this gene limited the accurate identification of SNPs using this method to the first 14 exons.

Each EST which aligned with the consensus was analyzed for the presence of potential SNPs by using the SEAN software package [48]. At least two ESTs must have the same base pair mismatch to be considered a presentable potential SNP.

### Generation of constructs

All polypeptide constructs were based on a previously well-characterized elastin-like polypeptide (ELP), EP20–24–24 [13]–[17]. This ELP consists of exons 20–21–23–24–21–23–24 of the human tropoelastin elastin gene. Primers used to introduce the desired mutations are listed below:

Primer 1: Ex-20-1G1S-BamHI

CGCGGATCCATGTTTCCCGGCTTTGGTGTCGGAGTCGGAGGTATCCCTGGAGTCGCAGGTGTCCCTAGTGTCGGAGGTGTTCC

Primer 2: Ex-20-3G3S-BamHI

CGCGGATCCATGTTTCCCGGCTTTGGTGTCGGAGTCGGAGGTATCCCTGGAGTCGCAGGTGTCCCTAGTGTCGGAGGTGTTCCCAGCGTCGGAGGTGTCCCGTCAGTTGGCATTTCC

Primer 3: Ex-23-1K1R-PpuMI

AGTGGGGACCCCAGCAGCTGCAGCTGCTAGAGCAGCCGCCAAAGCC

Primer 4: Ex-21 Apa

CGATTGGGCCCGAAGCTCAGGCAGCAGCTG

Primer 5: Ex-24 EcoRI

CTGCCTAGGGAATTCCTAAGGGCCAATCGCGGGAG

Primer 6: Ex-24 Apa

GCTTCGGGCCCAATCGCGGGAGCCAC

Primer 7: Ex-20 BamHI

CTGCTAGGGGGATCCATGTTTCCCGGCTTT

EP20–24–24 – [1G/1S] and EP20–24–24 – [3G/3S] were generated by PCR amplification of an EP20–24–24 template using upstream primers 1 and 2, respectively, and downstream primer 5. The resulting products were cut with the restriction enzymes BamHI and EcoRI. Since the EP20–24–24 construct includes two copies of exon 24, and therefore contains two EcoRI restriction sites, digestion leads to the production of both a smaller product corresponding to exons 20–21–23–24, and a larger product corresponding to exons 20–21–23–24–21–23–24. Gel electrophoresis of the digested products allowed the isolation and purification of the larger product.

Constructs EP20–24–24 – [1K/R] and EP20–24–24 – [2K/R] were generated by a two step process. The first step introduced the lysine to arginine mutation into a construct consisting of exons 20–21–23–24. This was accomplished by PCR with primers 3 and 5 on a template of exons 20–21–23–24 yielding an intermediate consisting of exons 23–24 containing the K to R mutation and with a PpuMI site at the 3′ end of exon 23 and an EcoRI site at the 5′ end of exon 24. (PpuMI-23[K/R]-24-EcoRI). An unmutated construct containing exons 20–21–23–24 was then digested with BamHI and PpuMI to produce an exon 20–21 intermediate, which was ligated to PpuMI-23[K/R]-24-EcoRI to produce an exon 20–21–23–24 construct containing the K to R mutation in exon 23 (exons 20–21–23[K/R]–24).

Next, using PCR with primers 6 and 7 and an unmutated template of exons 20–21–23–24, a construct consisting of exons 20–21–23–24 was generated with an ApaI site at the 3′ end and a BamHI site at the 5′ end (BamHI–20–21–23–24–ApaI). Then, PCR with primers 4 and 6 and a template consisting of exons 20–21–23[K/R]–24 was used to generate an intermediate consisting of ApaI–21–23[K/R]–24–EcoRI. This intermediate was digested with ApaI and ligated with the BamHI–20–21–23–24-ApaI construct to create the final construct for EP20-24-24 - 1[K/R].

The EP20–24–24 – 2[K/R] construct was generated using a similar method with the exception that EP20–24–24 – 1[K/R] was used with primers 6 and 7 to produce the construct to be ligated to the ApaI-21-23[K/R]-24-EcoRI intermediate.

For full-length human tropoelastin, all plasmids were constructed in a pGEX-2T expression vector (Amersham Biosciences) using the following primers:

Primer 8: BamHI-Ex2

TACGCGGATCCATGGGGGTCCCTGGGGCCATTC

Primer 9: hTE1074-BamHI

ACCTGGGATCCCAGCACCTGGGA

Primer 10: BsmI-Ex20-G

TGGAGGCATTCCTACTTACGGGGTTGGAGCTGGGGGCTTTCCCGGCTTTGGTGTCGGAGTCGGAGGTATCCCTGGAGTCGCAGGTGTCCCTGGTGTCGGAGGTGTTC

Primer 11: Ex36-Stop-EcoRI

CGTGCGAATTCCTACTTTCTCTTCCGGCC

Primer 12: BsmI-Ex20-3S

TGGAGGCATTCCTACTTACGGGGTTGGAGCTGGGGGCTTTCCCGGCTTTGGTGTCGGAGTCGGAGGTATCCCTGGAGTCGCAGGTGTCCCTAGTGTCGGAGGTGTTCCCAGCGTCGGAGGTGTCCCGTCAGTTGGCATTTC

hTE - [20:S]: The full-length tropoelastin cDNA flanked by EcoRI sites in pUC19-hTE (a gift of Dr. Robert Mecham, Washington University, St. Louis, MO) was inserted into the pGEX-2T vector, which had been digested with EcoRI. Note that this original cDNA contains the G to S polymorphism in domain 20. To put the coding sequence in frame and exclude the signal peptide sequence (exon 1), an approximately 1kb PCR product containing a BamHI site, start codon and tropoelastin exons 2 to 18 containing a natural BamHI site was generated using primers 8 and 9, with pUC19-hTE as template. The BamHI fragment from the pGEX-2T construct containing tropoelastin cDNA was removed and replaced with the 1 kb PCR product digested with BamHI to generate the hTE - [20:S] construct. The correct orientation and frame was confirmed by sequencing.

hTE - [20:G]: An approximately 1 kb PCR product containing tropoelastin exons 20 to 36 and a stop codon, incorporating the S to G mutation into domain 20, was generated using primers 10 and 11 with the hTE - [20:S] construct as template. The BsmI/EcoRI fragment containing tropoelastin exons 20 to 36 was removed from the hTE - [20:S] plasmid and replaced with the 1 kb PCR product digested with BsmI/EcoRI to generate the hTE - [20:G] construct.

hTE - [20:3S]: Using a similar approach, an approximately 1 kb PCR containing tropoelastin exons 20 to 36 and a stop codon, incorporating the three G to S mutations into domain 20, was generated using primers 11 and 12 with the hTE - [20:S] construct as template. The BsmI/EcoRI fragment containing tropoelastin exons 20 to 36 was removed from the hTE - [20:S] plasmid and replaced with the 1 kb PCR product digested with BsmI/EcoRI to generate the hTE - [20:3 S] construct.

In all cases PCR reactions were carried out using a HotStar HiFidelity PCR kit (Qiagen, Mississauga, Canada). Template and primer concentrations and PCR conditions were used as suggested by the manufacturer. PCR products were purified using a QIAEX II gel extraction kit (Qiagen) and ligation was performed using a Rapid DNA ligation kit (Fermentas, Burlington, Canada). All oligonucleotide primers were synthesized by Integrated DNA Technologies (Coralville, Iowa), and construct sequences were confirmed using facilities provided by The Centre for Applied Genomics at The Hospital for Sick Children.

### Protein expression and purification

Procedures for the recombinant expression and isolation of ELPs and tropoelastin variants from pGEX-2T vectors were as described previously [13], [14], [16], [17]. Briefly, the ELPs and tropoelastin variants are separated from the GST fusion protein by digestion with cyanogen bromide in 70% formic acid. This takes advantage of the fact that, unlike most other proteins, ELPs and tropoelastins contain no internal methionine residues as sites of cleavage for this reagent. After dialysis to remove small, non-elastin polypeptide fragments, the ELPs and tropoelastins were purified by ion exchange chromatography on Sepharose SP (Amersham Biosciences) followed by reverse-phase HPLC using a Jupiter 10 µm C4 200 Å column (Phenomenex, Torrence CA). Samples were then lyophilized and stored dry until use. Predicted molecular weights of ELPs were confirmed by mass spectrometry (Advanced Protein Technology Centre, Hospital for Sick Children).

### Circular dichroism (CD) spectroscopy

ELPs were dissolved in water to a concentration of 10 µM, as determined by the ratio of ultraviolet absorbance of the polypeptide in solution at 215 nm and 225 nm [49]. CD spectra were obtained step-wise in triplicate at 25°C using an AVIV 62DS spectrometer.

### Self-assembly behaviour

Coacervation experiments were performed using a Shimadzu UV-2401PC UV spectrophotometer (Mandel Scientific, Guelph, ON), equipped with temperature (Varian, Victoria, Australia), and stir bar controllers, using methods previously described [15], [26]. Briefly, ELPs were dissolved to a concentration of 25 µM in 50 mM Tris buffer, pH 7.5, containing 1.5 M NaCl. Solutions were placed into a quartz cuvette with a stir bar, inserted into the sample cell of the spectrophotometer and equilibrated for 5 min at a temperature approximately 5°C below the expected coacervation temperature. The solution temperature was then increased at a rate of 1°C per minute at a stirring rate of 1000 rpm, holding the temperature at 5°C above the coacervation temperature. Absorbance was monitored at 440 nm, recorded automatically at 18 sec intervals, and coacervation was detected as the onset of turbidity, appearing as a rapid increase in absorption. The coacervation temperature (Tc) was measured as the temperature at the onset of the increase in absorbance [15], [26].

### Fabrication of materials from ELPs and tropoelastin variants

Lyophilized ELPs and tropoelastins (5 mg) were dissolved in 370 µL of 0.15 M sodium borate buffer, pH 8.0, and stored overnight at 4°C. The solution was transferred to a 1 cm ×1 cm glass-bottomed well, and coacervation was induced by the addition of 80 µL of 5.0 M NaCl, with gentle mixing. This was followed by the addition, with gentle mixing, of 50 µL of 1001mM genipin (Challenge Bioproducts, Yun-Lin Hsien, Taiwan R.O.C.) dissolved in ethanol. Genipin is an extract from *Gardenia jasminoide* which has been used to crosslink a variety of proteins through side chains of lysine residues [18], [27]–[29]. The plates were immediately centrifuged at 3200 rpm for 2.5 min to deposit the coacervate onto the bottom of the well, holding the temperature above the coacervation temperature, followed by incubation overnight at 37°C. After incubation, the sheet of crosslinked material was removed from the glass-bottomed well by gentle flushing with water and stored in water at 4°C until use for mechanical testing.

### Measurement of tensile properties of materials fabricated from ELPs and tropoelastin variants

Tensile properties were measured in water at room temperature using a Biosyntech Mach-1 test apparatus (Montreal, QC) with a 1000 g load cell. After sample mounting, a photograph with scale bar was taken to record the initial length of the sheet between the grips (*L*_0_). For all tests the rate of extension was 103.6 µm/s. For measurements of % energy loss, the sample was pre-conditioned with three cycles of loading to an extension of 1.5 *L*_0_ followed by unloading at the same rate. Percent energy loss was then calculated from the fourth cycle of the load-extension data as:
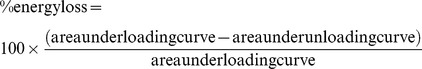



After three preconditioning cycles as described above, % stress relaxation was determined by loading the sample to 1.5 *L*_0_ and measuring the percent decline in load to maintain that extension over a period of 500 s. Finally, again after three preconditioning cycles as described above, the sample was loaded to failure at an extension rate of 103.6 µm/s. After failure, the portion of the material between the grips was removed and the dry weight determined by amino acid analysis (Advanced Protein Technology Centre, Hospital for Sick Children).

Load-extension data was normalized for comparison between samples. Strain was calculated as:
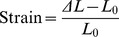



Elastic modulus, strain at break, % energy loss and % stress relaxation were reported for all samples. The elastic modulus of the sample was measured as the slope of the stress-strain curve. Stress is normally expressed as the load divided by the cross-sectional area of the sample. However, for these samples, the cross-sectional area was taken as the dry weight of the sample divided by *L*_0_. This measure of cross-sectional area reflects the amount of material bearing the load in the cross-section, and corrects for any variations in density of the material between samples [18]. Modulus was therefore calculated as: